# Analysis of genetic polymorphisms and tropism in East African *Leishmania donovani* by Amplified Fragment Length Polymorphism and kDNA minicircle sequencing

**DOI:** 10.1016/j.meegid.2018.07.016

**Published:** 2018-11

**Authors:** Hanan T. Jaber, Asrat Hailu, Francine Pratlong, Patrick Lami, Patrick Bastien, Charles L. Jaffe

**Affiliations:** a Department of Microbiology and Molecular Genetics, Kuvin Centre for the Study of Infectious and Tropical Diseases, IMRIC, Hebrew University - Hadassah Medical School, Jerusalem, Israel; b School of Medicine, College of Health Sciences, Department of Microbiology, Immunology & Parasitology, Addis Ababa University, Addis Ababa, Ethiopia; c Department of Parasitology-Mycology, National Reference Centre for Leishmanioses, Academic Hospital of Montpellier, France; d University of Montpellier, CNRS 5290/IRD 224 “MiVEGEC”, Montpellier, France

**Keywords:** Amplified Fragment Length Polymorphism, HIV-visceral leishmaniasis co-infections, Post-kala azar dermal leishmaniasis, *Leishmania donovani*, Genetic polymorphism, kDNA minicircle sequence

## Abstract

Visceral leishmaniasis (VL), the most severe form of leishmaniasis, is caused by *Leishmania donovani*. In addition to fatal VL, these parasites also cause skin diseases in immune-competent and -suppressed people, post-kala azar dermal leishmaniasis (PKDL) and HIV/VL co-infections, respectively. Genetic polymorphism in 36 Ethiopian and Sudanese *L. donovani* strains from VL, PKDL and HIV/VL patients was examined using Amplified Fragment Length Polymorphism (AFLP), kDNA minicircle sequencing and Southern blotting. Strains were isolated from different patient tissues: in VL from lymph node, spleen or bone marrow; and in HIV/VL from skin, spleen or bone marrow. When VL and PKDL strains from the same region in Sudan were examined by Southern blotting using a DNA probe to the *L. donovani* 28S rRNA gene only minor differences were observed. kDNA sequence analysis distributed the strains in no particular order among four clusters (A – D), while AFLP analysis grouped the strains according to geographical origin into two major clades, Southern Ethiopia (SE) and Sudan/Northern Ethiopia (SD/NE). Strains in the latter clade were further divided into subpopulations by zymodeme, geography and year of isolation, but not by clinical symptoms. However, skin isolates showed significantly (*p* < 0.0001) fewer polymorphic AFLP fragments (average 10 strains = 348.6 ± 8.1) than VL strains (average 26 strains = 383.5 ± 3.8).

## Highlights

• Strains from VL, post kala-azar dermal leishmaniasis and HIV/VL co-infection patients examined
• Bone marrow and spleen strain of same HIV/VL patient different
• AFLP, kDNA sequencing and Southern blotting fail to group strains on basis of clinical symptoms
• AFLP clades are divided by geography, not clinical symptoms
• Skin isolates show fewer polymorphic AFLP fragments than visceral strains

## Graphical abstract

**Figure:**
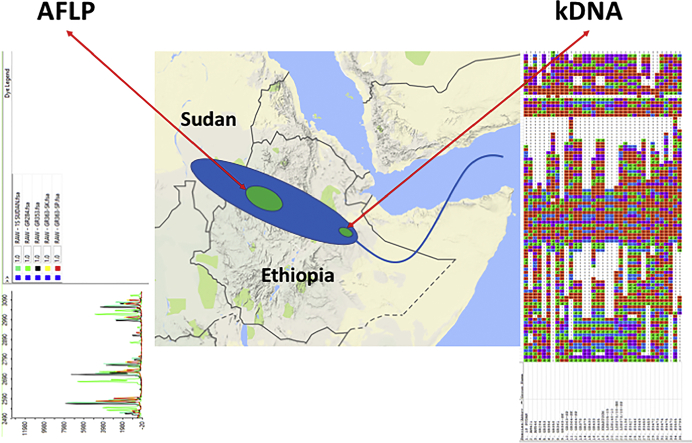

## 1 Introduction

The leishmaniases are vector-borne diseases caused by several species and subspecies of obligate intracellular parasites belonging to the genus *Leishmania*. Diseases caused by these parasites are endemic in tropical and subtropical regions of both the Old and New World. Based on the clinical pattern of disease they are classified into three major forms: cutaneous, mucocutaneous and visceral leishmaniasis (Bañuls et al., 2011). Cutaneous leishmaniasis is the mildest form of the disease and is typified by localized skin lesions at the site of the sandfly bite that generally heal spontaneously with significant scarring. In mucocutaneous leishmaniasis, the parasites metastasize from the original lesion to the naso-pharynx region where massive tissue destruction may occur resulting in extensive disfigurement. This form of disease does not self-cure nor does it respond well to drug treatment. Finally, visceral leishmaniasis (VL), the most severe form of disease, has a high mortality rate if not diagnosed and treated efficaciously. VL is caused by *Leishmania donovani* and *L. infantum*. These parasites preferentially infect the liver, spleen and bone marrow. The estimated annual number of new cases of VL worldwide is about 200,000–400,000 (Alvar et al., 2012).

Post kala-azar dermal leishmaniasis (PKDL) is a skin-condition that can develop in VL patients either during or after treatment but may also occur in previously asymptomatic patients with no history of VL. PKDL is usually chronic and exhibits various pathologies that include macular, papular or nodular lesions. It is restricted to areas where *L. donovani* is prevalent, and occurs in approximately 5–10% of Indian, and 50% of Sudanese VL cases. It generally appears following recovery from VL within 0.5–6 years in India and Nepal, or 0–13 months in East Africa (Mukhopadhyay et al., 2014; Zijlstra, 2014; Zijlstra et al., 2003). In Ethiopia, PKDL is endemic in the northern focus (Metema/Humera), which is an extension of the endemic region in Eastern Sudan (Zijlstra et al., 2003). Approximately, 14% of the VL patients develop PKDL within 6 months after cure, and PKDL is more prevalent in HIV-positive patients (27.3%) than in HIV-negative patients (13.3%) (Ritmeijer et al., 2001).

Even though the same species, *L. donovani*, causes both VL and PKDL, little is known regarding the factors, both host and parasite, that regulate these different pathologies. In addition to host parameters, parasite genetic determinants may play a role in the survival and persistence of *Leishmania* in different host organs, skin versus viscera. A few reports have compared Indian VL and PKDL strains. In one study, polymorphism in the 28S rRNA gene locus was postulated to account for the difference in tissue tropism (Sreenivas et al., 2004). PKDL parasites were shown to have higher expression levels of surface proteins e.g., gp63 and gp46 (Salotra et al., 2006). Southern blotting using a ß-tubulin probe identified different banding patterns for PKDL and VL isolates (Dey and Singh, 2007). In addition, PKDL isolates showed lower aquaglyceroporin (AQP1) expression compared to VL isolates (Mishra et al., 2013).

*Leishmania*, especially *L. infantum* and *L. donovani*, are opportunistic pathogens. HIV/*Leishmania* co-infections have been reported in 35 countries, and currently account for 2–9% of all VL cases in endemic countries (Alvar et al., 2008). In Sudan, HIV prevalence is low (1.3%), so few cases of HIV/VL co-infection have been reported (Diro et al., 2014; Zijlstra, 2014). The rate of HIV/VL co-infection in Ethiopia is between 15 and 30%, with the most important foci found in the Metema / Humera regions where 60% of the total cases of VL occur (Haile and Anderson, 2006; Hurissa et al., 2010; Lyons et al., 2003). In AIDS patients, *Leishmania* are found in organs they do not normally invade in immunocompetent people, and skin lesions are commonly described in HIV/VL patients infected with *L. donovani* or *L. infantum* (Zijlstra, 2014).

One of the central questions in the *Leishmania* research is why some species only cause cutaneous disease, whereas others cause primarily visceral disease? In the case of the *L. donovani* complex, parasites reside in various internal organs (liver, spleen, lymph nodes, bone marrow), as well as the skin of PKDL and HIV/VL patients (Zijlstra et al., 2003). In recent years, our understanding of the parasite genetic determinants of tropism has improved, and several reports suggest that specific parasite genes such as the A2 family, LinJ.28.0340, LinJ.15.0900, and a ras-like RagC GTPase are involved (Zhang et al., 2014).

Despite these new findings, the underlying biology of tropism and the development of PKDL still remain elusive (Mukhopadhyay et al., 2014; Zijlstra, 2014; Zijlstra et al., 2003). Little is known about the factors, parasite or host, which cause a shift in the infection site from the viscera to the dermis resulting in PKDL. Furthermore, it is not known whether the parasites in PKDL lesions represent residual parasites from the original infection or are due to re-infection. In the former case, the shift could be due to the altered immune status of the host (Zijlstra, 2016).

East Africa is an important focus of VL where it is endemic in eight countries, with an estimated incidence between 29,400–56,700 cases per year (Alvar et al., 2012; Consortium, 2010). Most of the cases are found in Sudan, South Sudan and Ethiopia (Alvar et al., 2012). In Ethiopia six endemic areas with an estimated annual incidence of 4000 cases have been identified (Haile and Anderson, 2006). In Sudan, VL occurs in different foci: Kapoeta near the Kenyan border, the Western Upper Nile (Seaman et al., 1996), and a wide belt extending from the White Nile eastward to the Sudanese-Ethiopian border (El-Hassan et al., 1995; El-Safi et al., 2002; Elnaiem et al., 2003); and extending into the Metema - Humera focus in Ethiopia (Consortium, 2010).

Amplified Fragment Length Polymorphisms (AFLP) is a PCR technique that allows the comparison and identification of genetic polymorphisms randomly distributed across the entire genome and has been proven as a powerful technique to infer phylogenetic relationships among many organisms. This method allows the simultaneous screening of multiple markers randomly distributed over the entire genome and can be applied to organisms where genomic sequences are not available. As such it offers a good alternative to whole genome sequencing (Meudt and Clarke, 2007; Odiwuor et al., 2011), and can be used to identify unique regions that may be associated with phenotypic differences among organisms.

In *Leishmania*, AFLP has been used to examine parasite drug resistance (Kumar et al., 2009), differences between species causing VL and CL (Kumar et al., 2010), and taxonomy of the *L. Viannia* subgenus (Odiwuor et al., 2012; Restrepo et al., 2013). Odiwuor et al. (Odiwuor et al., 2011) employed twelve specific primer pairs, six of which were used in this study, to analyze genetic diversity among 29 strains belonging to the *L. donovani* complex (11 *L. donovani* and 18 *L. infantum*) from East Africa, Indian subcontinent, Europe and South America. A total of 852 markers, 52% polymorphic, were used. Three major clusters were identified: one containing East African (Sudan and Ethiopia) strains, a second containing mixed Kenyan and Indian isolates, and the third comprised of *L. infantum* strains. These results were consistent with molecular analysis of the same isolates using other techniques such as MLMT, MLST and MLEE (Baleela et al., 2014; Downing et al., 2011, 2012; Kuhls et al., 2007; Lukes et al., 2007; Mauricio et al., 2006; Ochsenreither et al., 2006; Zemanová et al., 2007) showing that AFLP provides a reliable reproducible method for analyzing genetic diversity.

Mitochondrial or kinetoplast DNA (kDNA) in trypanosomatids consists of a network of 10–20 maxicircles (20–40 kbp in size) and ~10,000 minicircles (<1 kbp in size) (Jensen and Englund, 2012). The maxicircle encodes mitochondrial genes and rRNA, while minicircles, with the exception of a few small regions involved in their replication, are highly polymorphic and encode guide RNAs that function in the process of mRNA editing of the mitochondrial genes (Jensen and Englund, 2012). Minicircle DNA sequences are highly heterogeneous and undergo rapid evolution (Rogers and Wirth, 1987). kDNA minicircle analysis, by either PCR-RFLP or sequencing, has been shown to be highly discriminatory, even among strains that appear genetically homogenous by other techniques (Bhattarai et al., 2010; Cortes et al., 2006; Cuervo et al., 2004; Laurent et al., 2007). Some reports have shown that kDNA genotypes are associated with host species origin (Alonso et al., 2010; Oryan et al., 2013a) and clinical disease (Oryan et al., 2013b).

In this work genetic polymorphism was examined in *L. donovani* strains isolated from patients with different clinical symptoms (VL, PKDL and HIV/VL co-infections) and tropism (lymph node, spleen, bone marrow and skin isolates) using two methods, AFLP analysis and kDNA minicircle sequencing. The majority of strains originated from similar biotopes in Northern Ethiopia (NE) and Eastern Sudan (SD) where the parasite is transmitted by *P. orientalis* (Elnaiem, 2011). Most of the SD strains were isolated in a single village, Barbar El Fugara, not far from the Ethiopian border (El-Safi et al., 2002; Elnaiem et al., 2003) during the large outbreak that occurred in Gedarif state between 1996 and 1999. This epidemic has spread to Eritrea and northwest Ethiopia, and the NE strains were isolated from a VL focus near the border with SD (Consortium, 2010). VL predominantly affects young male migrant workers in NE (Alvar et al., 2007; Mengesha and Abuhoy, 1978; Ritmeijer et al., 2006), whereas in SD the majority of cases were children (Collin et al., 2004; El-Safi et al., 2002; Elnaiem et al., 2003). Polymorphisms and subtle differences in nuclear DNA and kDNA minicircles among the strains were explored.

## 2 Materials and methods

### 2.1 Parasites and DNA extraction

A total of 37 *L. donovani* strains from Ethiopia (*n* = 22) and Sudan (*n* = 15) were used in this study. Nine strains are from HIV/VL co-infected patients. Eight strains are from PKDL patients in Sudan. A description of the *L. donovani* parasites used is given in Table S1. *L. major* (MHOM/IL/81/Friedlin) was used as an outlier for the AFLP studies. All parasites were grown at 26 °C in complete M199 medium (pH 6.8) supplemented with 10% fetal calf serum (Zackay et al., 2013). Leishmanial DNA was extracted using the phenol-chloroform method (Schonian et al., 2003). Strain MHOM/SD/97/LEM3472 (SD9) was cloned using the hanging drop methodology as previously described by (Garin et al., 2002).

### 2.2 Ethics statement

This study was reviewed and approved by the Institutional Review Board (IRB), Medical Faculty, Addis Ababa University. Written informed consent was obtained from each study participant.

### 2.3 Parasite characterization

**Fig. S1:**
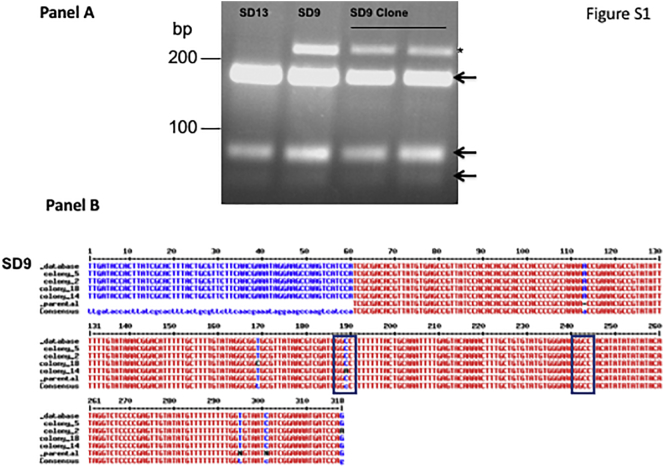
Internal transcribed spacer 1 (ITS1)–PCR analysis of Sudanese *Leishmania donovani* strain SD9 (LEM3472). Panel A – Parasites were cloned (Garin et al., 2002), and ITS-1–PCR RFLP carried out (Schonian et al., 2003). Ethidium-bromide stained agarose gel showing the parent strain SD9 (MHOM/SD/97/LEM3472) with an extra band at 239 bp*, and another Sudanese strain SD13 (MHOM/SD/98/LEM3570) displaying typical HaeIII digestion patterns (189, 77 and 51 bp arrows). Panel B - Sequence alignment of ITS1–PCR region of the Sudanese PKDL strain MHOM/SD/97/LEM3472 (SD9). The PCR products were cloned into pGEM-T Easy and colonies picked for sequencing, and DNA sequences were compared using MultAlin (http://sacs.ucsf.edu/cgi-bin/multalin.py). Sequences examined: Genebank – SD9_database (accession number emb|AJ634370.1|), Parental strain – SD9_parental (this study), and SD9 amplicons subcloned into pGEM –T Easy - colony_5, _2, _14 and _18 (this study). In colony 14 a single nucleotide point mutation (C189A) is present eliminating the HaeIII restriction site (GGCC) resulting in an extra 239 bp product. Additional single nucleotide point mutations, colony 18 (T169C) and colony 2 (G318A) were noted suggesting that different ITS-1 sequences exist in the tandem repeats (Downing et al., 2011; Gelanew et al., 2010a) of the ITS1 regions between the SSU rRNA and the 5.8S genes on chromosome 27.

Initial species characterization was carried out by several methods including: Internal Transcribed Spacer-1 (ITS-1) PCR followed by restriction fragment length polymorphism (RFLP) (Schonian et al., 2003), cpbE/F - PCR (Zackay et al., 2013), k26 – PCR (Zackay et al., 2013) and/or multilocus enzyme electrophoresis (MLEE) (Dereure et al., 2003; El-Safi et al., 2002; Pratlong et al., 2001). The ITS-1 PCR product of one strain, SD9, which gave an atypical RFLP pattern (Fig. S1, panel A), was purified using Wizard SV Gel and PCR Clean-Up System (Promega®) and cloned into pGEM-T Easy Vector System (Promega®). Colonies (*n* = 4) were picked and the inserts were sequenced. In addition, the PCR product of the parental strain was sequenced. These sequences (Fig. S1, panel B), and the sequence previously deposited in GenBank (Accession No. emb|AJ634370.1|) were compared using MultAlin (Corpet, 1988).

### 2.4 kDNA minicircle genotyping

PCR was performed using the primer pair: Uni21 (5′-GGG GTT GGT GTA AAA TAG GG) and Lmj4 (5′-AAA ATG AAC GGG ATT TCT GC) (Anders et al., 2002). *Leishmania* DNA was added to a reaction mixture (25-μl final volume) containing High Yield ready mix (Syntezza Bioscience®) and primers (2 μM). The PCR conditions were as follows: initial denaturation 95 °C 5 min; 30 cycles of denaturation 95 °C 20 s, annealing 63 °C 25 s and extension 72 °C 40 s; final extension in the last cycle was 6 min 72 °C. Amplicons were analyzed by 2% agarose gel electrophoresis, and the gels stained with ethidium bromide (0.3 μg/ml). PCR products were isolated using the Wizard SV gel and PCR Clean-Up System (Promega®) and cloned into pGEM-T Easy vector (pGEM-T Easy Vector System, Promega®) according to the manufacturer's instructions. DH5α cells were transformed using heat shock for 45 s at 42 °C and grown in LB medium. Colonies containing inserts were picked and added directly to a PCR mix containing Dream Taq PCR master mix (MBI Fermentas®) in a total volume of 50 μl containing 0.5 μM of the pGEM-T Easy Vector primers: SP6: 5′-TAT TTA GGT GAC ACT ATA G-3′, and T7: 5′-TAA TAC GAC TCA CTA TAG GG-3′. PCR conditions for insert amplification were: initial denaturation 5 min at 95 °C followed by 35 cycles of 30 s at 95 °C, 40 s at 56 °C and 45 s at 72 °C with a final elongation step of 10 min at 72 °C. PCR products, one colony per strain, were sequenced directly. The sequences were aligned using Muscle algorithm with gaps, and the phylogenetic tree was built using Maximum Likelihood method (ML). The best model, Hasegawa-Kishino-Yano model (Hasegawa et al., 1985) with Gamma-distributed rates (5 categories, +G = 1.6955) plus Invariant Sites (+I = 0.1725) was determined using the “Find best DNA/protein model (ML) function” in MEGA 7.0.2 (Kumar et al., 2016). An essentially identical tree was obtained using the Tamura-Nei model +G and + I (Tamura and Nei, 1993). Nearest-Neighbor-Interchange (NNI) was used as the ML Heuristic Method. Initial tree(s) for the heuristic search were obtained automatically by applying Neighbor-Join and BioNJ algorithms to a matrix of pairwise distances estimated using the Maximum Composite Likelihood (MCL) approach, and then selecting the topology with superior log likelihood value (−4468.83). The analysis involved 35 nucleotide sequences. All positions containing gaps and missing data were eliminated. There was a total of 287 positions in the final dataset.

### 2.5 Southern blotting

A DIG-dUTP (Roche Applied Science®) labeled DNA probe (Ld28S) to the ζ and ε subunit regions of the *L. donovani* (MHOM/SD/00/Khartoum) 28S rRNA gene (accession no. AF115465) was prepared by PCR, and used for Southern blotting of parasite DNAs. The complete protocol is described in the supporting material.

### 2.6 Amplified Fragment Length Polymorphism (AFLP) fingerprinting

AFLP was carried out as previously described (Odiwuor et al., 2011) with some modifications.

#### 2.6.1 Restriction digestion and ligation of adaptors

Following digestion with TaqI (10 U, New England Biolabs®), the mixture was cooled on ice, and then 10 U PstI HF (New England Biolabs®) was added and the reaction incubated for an additional 1 h at 37 °C. Subsequently, the pre-annealed specific adaptors (Syntezza Bioscience®) (Table S2) were ligated to the genomic DNA fragments using T4 DNA ligase (1 U, MBI Fermentas®). Ligation was done at 22 °C for 3 h, and the resulting RL products used as templates in AFLP PCR without heat inactivation of endonucleases.

#### 2.6.2 AFLP PCR

Adaptor-ligated fragments were subjected to two successive PCR reactions. First, pre-selective PCR was done using Dream Taq PCR master mix (MBI Fermentas®) in a total volume of 50 μl containing 5 μl of the four-fold diluted RL products and 0.5 μM of the primers PstI+0 and TaqI+0 (Table S2). The PCR conditions were: initial denaturation 5 min at 95 °C followed by 20 cycles of 20 s at 95 °C, 30 s at 56 °C and 1 min at 72 °C with a final elongation step of 15 min at 72 °C. Pre-selective amplification products were four folded diluted in water and used as templates for a second, selective, PCR. Selective PCR amplification was carried out using JumpStart™ Taq DNA Polymerase with MgCl_2_ (Sigma Aldrich®) in a total volume of 50 μl using different pairs of labeled and non-labeled primers (Table S2). Each reaction mixture contained: 1× PCR buffer, MgCl_2_ (2.5 mM), 0.2 mM each dNTP (Larova GmbH®), 1 μM each selective primer pair (Table S2), 2.5 U Taq DNA polymerase, and 5 μl of the diluted pre-selective amplification products. The selective PCR conditions were as follows: the DNA was denatured for 5 min at 95 °C then subjected to nine cycles: denaturation for 20 s at 95 °C, annealing for 30 s at 66 °C and elongation 3 min at 72 °C. The annealing temperature was lowered by 1 °C in each cycle. Following this, the PCR was subjected to 20 cycles: denaturation at 95 °C for 20 s, annealing at 56 °C for 30 s and elongation 72 °C for 3 min; the last elongation step lasted for 45 min at 72 °C.

#### 2.6.3 Fragment and data analysis

Labeled selective AFLP PCR fragments were detected by capillary gel electrophoresis (CGE). PCR products (1 μl) were combined with 0.04 μl of Genescan 500 LIZ Size Standard (Applied Biosystems®) and HiDi formamide (10 μl, Applied Biosystems®). CGE was carried out on an ABI PRISM™ 3730xl DNA Analyzer (50 cm POP 7 polymer capillary and a G5 filter). The raw data were collected with Peak Scanner Software version 1.0. (Applied Biosystems®) and analyzed using the following programs: optiFLP (Arthofer et al., 2011), tinyFLP (Arthofer, 2010), tinyCAT (Arthofer, 2010) and SplitsTree 4 (Huson and Bryant, 2006). The AFLP scoring parameters were optimized using optiFLP (Arthofer et al., 2011). Nine different parameters were examined and individually set for each primer pair in order to identify genuine bands and eliminate background noise. Of the nine parameters, minimum peak height and minimum peak size were determined by examining the negative control profile for a sample lacking DNA. The minimum peak height was set slightly above the highest value seen in the negative control profile, while minimum peak size was designated as bands showing at least 3-fold increase in signal to noise ratio relative to the negative control. Peaks ranging from 48 bp to a maximum of 350–500 bp, depending on the primer pair, were included in the analysis. tinyFLP (Arthofer, 2010) generates statistical data for all the bands allowing the user to identify: 1) samples with poor performance; 2) monomorphic bands that are present in all the samples and have no phylogenetic value, and 3) artifacts e.g., bands that appear in only one sample. Bins with rare alleles present only in one sample, as well as monomorphic bands that lack phylogenetic information, were removed from further analysis. tinyCAT was used to concatenate the binary allele matrices (BAM) for each sample, using the 6 different primer combinations, into one combined matrix (Arthofer, 2010). This combined BAM was used in all subsequent applications for phylogenetic analysis (Arthofer et al., 2011). A network tree containing 37 strains was generated using the program SplitsTree 4 (Huson and Bryant, 2006) by using Neighbor-net from 411 data points i.e., AFLP peaks (Bryant and Moulton, 2004).

Validation of the AFLP data and the phylogenetic tree derived by combined analysis using 6 primer pairs was carried out by bootstrapping (Holland et al., 2008). The reliability of AFLP markers was assessed by the error rate, which is the ratio of the number of mismatched loci to the total number of loci (Bonin et al., 2004; Ley and Hardy, 2013). Mismatches can be calculated within duplicates across all loci or for each locus across all duplicates (Bonin et al., 2004; Bonin et al., 2007; Zhang and Hare, 2012). Replicate error rate due to biological or technical causes was used to calculate the mismatch error rate using the formula stated in (Bonin et al., 2004; Bonin et al., 2007; Holland et al., 2008).

Technical error was evaluated using two approaches: 1) by performing duplicate selective AFLP PCRs using the same pre-selective PCR product obtained from two strains MHOM/SD/1997/LEM3459 (SD8a and SD8b), and MHOM/ET/2009/GR353 (NE2a and NE2b), and 2) by performing the complete AFLP protocol on 25 different parasite genomic DNA samples using one selective primer pair (PstI+AG / TaqI+AA). The amplicon fragments were analyzed separately for each approach on individual capillary electrophoresis runs (Odiwuor et al., 2011). Biological error was determined by comparing, for one parasite strain NE11 (MHOM/ET/2010/GR363sp), the fragments generated after the AFLP PCR (pre-selective and selective PCR) procedure using pure DNA from three separate extractions. Only one set of selective PCR primers (PstI+AG / TaqI+AA) was tested, and all the products were analyzed by capillary gel electrophoresis at the same time (Odiwuor et al., 2011). Previous AFLP studies have shown that error rates, due to biological or technical origins, ranging from 2% to 5%, (Bonin et al., 2004; Bonin et al., 2007; Holland et al., 2008) are expected for this technique (Bonin et al., 2004). The calculated error due to biological or technical origins was 3.0%.

The concatenated BAM containing all the strains except *L. major* and two of the technical replicate control: SD8b and NE2b were analyzed using the software STRUCTURE (Pritchard et al., 2000) in three steps. Ten iterations were run for K1 to 10 after a burn-in of 100,000 simulations, using the options of admixture and correlated allele frequencies. The most suitable K number was calculated using Structure Harvester (Earl and Vonholdt, 2012) then the same analysis was run for another round on two (NE strains and SD MON-18 strains) of the 4 subpopulations, and K1 to 5/6 was checked using 10 iterations. A third round of analysis for SD MON-18 strains was performed using 10 iterations and K1 to 9.

## 3 Results

**Fig. 1:**
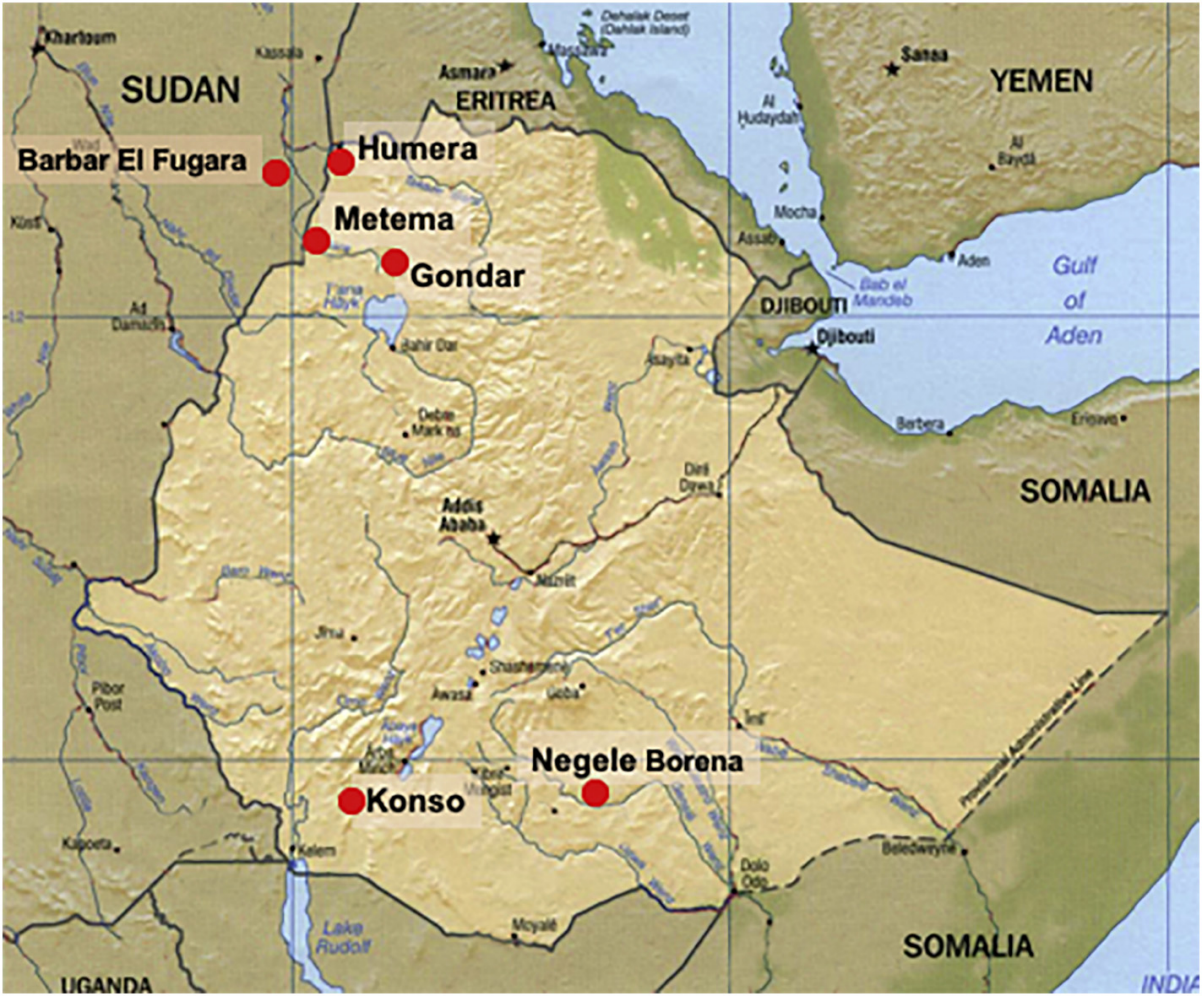
Map indicating the sites where the *Leishmania donovani* strains examined in this study were isolated in Sudan and Ethiopia. This figure was adapted from Gelanew et al., 2010b.

Genetic polymorphism of 33 *L. donovani* strains originating from the endemic region extending from eastern Sudan into northwestern Ethiopia (Fig. 1) was investigated by several techniques. Three strains from southern Ethiopia (SE), all from spleens, were included for comparison. Most Sudanese (SD) strains originated from the village Barbar El Fugara, while the majority of Ethiopian strains originated from the Metema - Humera region (Dereure et al., 2003; Gelanew et al., 2010b; Pratlong et al., 2001; Rougeron et al., 2011; Zackay et al., 2013) in northwestern Ethiopia. The Ethiopian strains are more recent in origin and were isolated from patients in 2009–2010, while most of SD strains were isolated 11–14 years earlier (Table S1). The parasites examined were isolated from patients with: VL, PKDL and HIV/VL co-infections. Strains were isolated from different organs including bone marrow (*n* = 6), spleen (*n* = 10), lymph nodes (*n* = 5) and skin (n = 10) (Dereure et al., 2003; Pratlong et al., 2001; Zackay et al., 2013). HIV infection was absent in the SD patients (Dereure et al., 2003). Eight of the skin strains were isolated from PKDL patients and the remaining two from HIV/VL patients. The strains examined also included three parasite pairs, isolated from two different tissues of HIV/VL patients: either spleen and skin (2 pairs) or spleen and bone marrow (1 pair).

All the SD strains were originally typed by MLEE analysis ((Dereure et al., 2003; Pratlong et al., 2001) and Table S1); however, prior to AFLP they were re-examined by ITS-1 PCR-RFLP (Schonian et al., 2003), cpb E/F - (Zackay et al., 2013) and k26–PCR (Zackay et al., 2013). All the SD strains showed PCR and RFLP products characteristic of *L. donovani*; and a 290 bp k26 PCR amplicon typical of parasites isolated in SD and Northern Ethiopia (NE) (Zackay et al., 2013). Characterization of the Ethiopian strains by ITS-1–PCR RFLP, cpb E/F –and k26-PCR has already been described (Zackay et al., 2013).

One isolate from a PKDL patient, SD9 (MHOM/SD/97/LEM3472), showed an extra 239-bp band, a pattern previously described in a strain isolated from an Ethiopian PKDL patient (Gelanew et al., 2010a) carrying a mutation in the HaeIII restriction site, in addition to the three bands normally observed for L. *donovani* following HaeIII digestion of the ITS-1–PCR product (Fig. S1, panel A). We ruled out the possibility that SD9 represents a mixture of several parasite variants by carrying out RFLP analysis on a clone, and sequencing of ITS1-PCR amplicons (Fig. S1, panel B).

### 3.1 Amplified Fragment Length Polymorphisms (AFLP) alleles

**Fig. 2:**
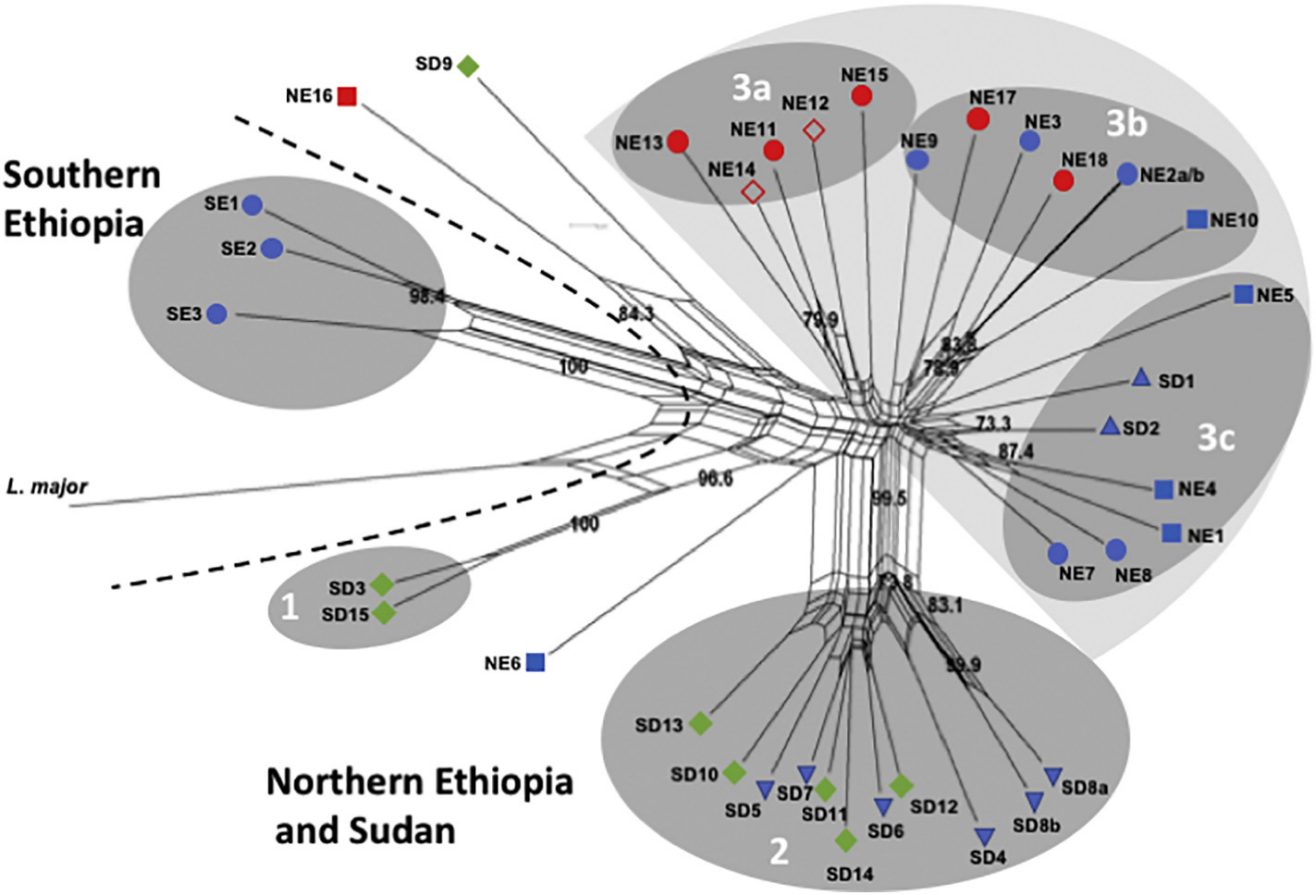
Neighbor-net splits graph derived from combined analysis of AFLP using six selective primers. Results were analyzed using optiFLP (Arthofer et al., 2011) and tinyFLP (Arthofer, 2010), and the binary matrices concatenated using tinyCAT (Arthofer, 2010). The tree above was built using Neighbor-net analysis of the concatenated binary matrix with Splitstree 4 (Huson and Bryant, 2006). The numbers above each branch indicate the bootstrap support in percentage from 1000 replicates. SD1 and SD2 are reference strains from Sudan that were isolated in 1943 and 1962, respectively. Strains NE2a and NE2b, as well as SD8a and SD8b, are technical duplicates. Selective PCRs for these strains were run in duplicate using the same pre-selective PCR product. Strain origin: Northern Ethiopia (NE) groups 3a, 3b and 3c, Southern Ethiopia (SE) group1, and Sudan (SD) groups 2 and 3c. Clinical presentation: VL – blue, HIV/VL co-infection – red, PKDL – green. Tissue source: skin isolates – both full and empty diamonds, spleen isolates - circles, lymph node -inverted triangle, bone marrow isolates - squares, and no information - pyramid. *Leishmania major* was included as an outlier. (For interpretation of the references to colour in this figure legend, the reader is referred to the web version of this article.)

AFLP analysis using six selective primer combinations (Taq + AA & PstI+AG, Taq + AC & PstI+AG, Taq + AA & PstI+GT, Taq + AC & PstI+TG, Taq + AG & PstI+TG and Taq + AC & PstI+CC; Table S2) was performed on 36 *L. donovani* strains from SD and Ethiopia, and *L. major* included as an outlier. These primer pairs were chosen because they gave the most intense PCR products and widest molecular weight range of products of all the 12 primer pairs examined in initial AFLP runs on six of the strains. Of the 456 AFLP alleles included in this study, 45 alleles (~10%) were monomorphic while the remaining 411 (90%) were polymorphic. A Neighbor-net network (Fig. 2) was built using the 411 polymorphic data points i.e., AFLP peaks. Strains GR353 (NE2a and NE2b) and LEM3459 (SD8a and SD8b) were used as internal controls. The selective PCR was performed in duplicate using the same pre-selective PCR product giving essentially identical replicates.

As can be seen in Fig. 2 strains from Southern Ethiopia (SE) group separately from the Northern Ethiopian and Sudanese (NE/SD) strains. The latter strains include parasites from VL (blue symbols), HIV/VL co-infected (red symbols) and PKDL (green symbols) patients, as well as two reference *L. donovani* strains (SD1 and SD2) isolated 66 and 48 years, respectively, prior to the other SD strains. Only 5 out of 33 NE/SD strains fail to cluster with the two main populations. These include two PKDL strains, SD3 and S15, from Barbar El Fugara that are found in group 1. Group 1 is strongly supported by a bootstrap value of 100%. These strains have the zymodemes, MON-82 and MON-30, respectively, and have been classified in the past by isoenzyme analysis as *L. archibaldi* and *L. infantum*, respectively (Dereure et al., 2003; Pratlong et al., 2013), though characterization using other molecular markers recommended classification as *L. donovani s.s* (Jamjoom et al., 2004; Lukes et al., 2007). They cluster differently from all the other SD strains. SD9 which has zymodeme MON-267 also fails to group with any of the SD strains examined. The zymodemes for the NE strains were not determined. The NE/SD strains were further divided into four groups. Group 2 contains all the MON-18 strains from Barbar El Fugara isolated in 1997/1998 (SD4 – SD8 and SD10 – SD14). This clade includes isolates from both VL (blue) and PKDL (green) patients. Finally, the two laboratory reference strains SD1 (Khartoum) and SD2 (1S Sudan) isolated in 1943 and 1962, respectively, clustered together with five NE strains (Group 3c) isolated in 2009–2010, rather than the SD strains from Barbar El Fugara. Most of the NE strains (16 out of 18) clustered in one large clade (group 3) that also contains the reference Sudanese strains mentioned above. The NE strains, isolated in 2009–2010, appear to be more polymorphic than the SD strains from Barbar El Fugara, and are distributed among several subpopulations. Interestingly, 5 out of 8 NE HIV/VL strains (NE11 – NE15) clustered together (group 3a), however four of these strains, NE11 - NE14, originated from different tissues, spleen (NE11 and NE13) or skin (NE12 and NE14), of only two patients. In a previous study on *L. donovani* genotypes in Ethiopia, identical multilocus microsatellite typing (MLMT) profiles were found for three different pairs of parasites, each pair originating from a single HIV/VL patient (spleen and skin or bone marrow and skin). These authors suggested that parasites in the skin of these patients disseminated from visceral tissues (Gelanew et al., 2011). Interestingly, NE15, the fifth strain in group 3a, also belongs to a matched pair isolated from one patient. However, in this case, NE15 (spleen) and NE16 (bone marrow) did not cluster together. These two isolates also differed in the size of their k26-PCR product. The amplicon for the spleen isolate is typical of NE strains (290 bp), while the amplicon for the bone marrow isolate is atypical for this region (410 bp) (Zackay et al., 2013). The remaining two HIV/VL strains (NE17 and NE18) isolated from spleens clustered in yet a third group (Fig. 2, group 3b). Interestingly, 5 out of 6 strains in group 3b were isolated from the spleen (NE2, NE3, NE9, NE17 and NE18). This group includes isolates from both VL and HIV/VL co-infected patients. The remaining five NE strains formed a third group (3c) that contains three bone marrow (NE1, NE4 and NE5) and two spleen isolates (NE7 and NE8).

**Fig. 3:**
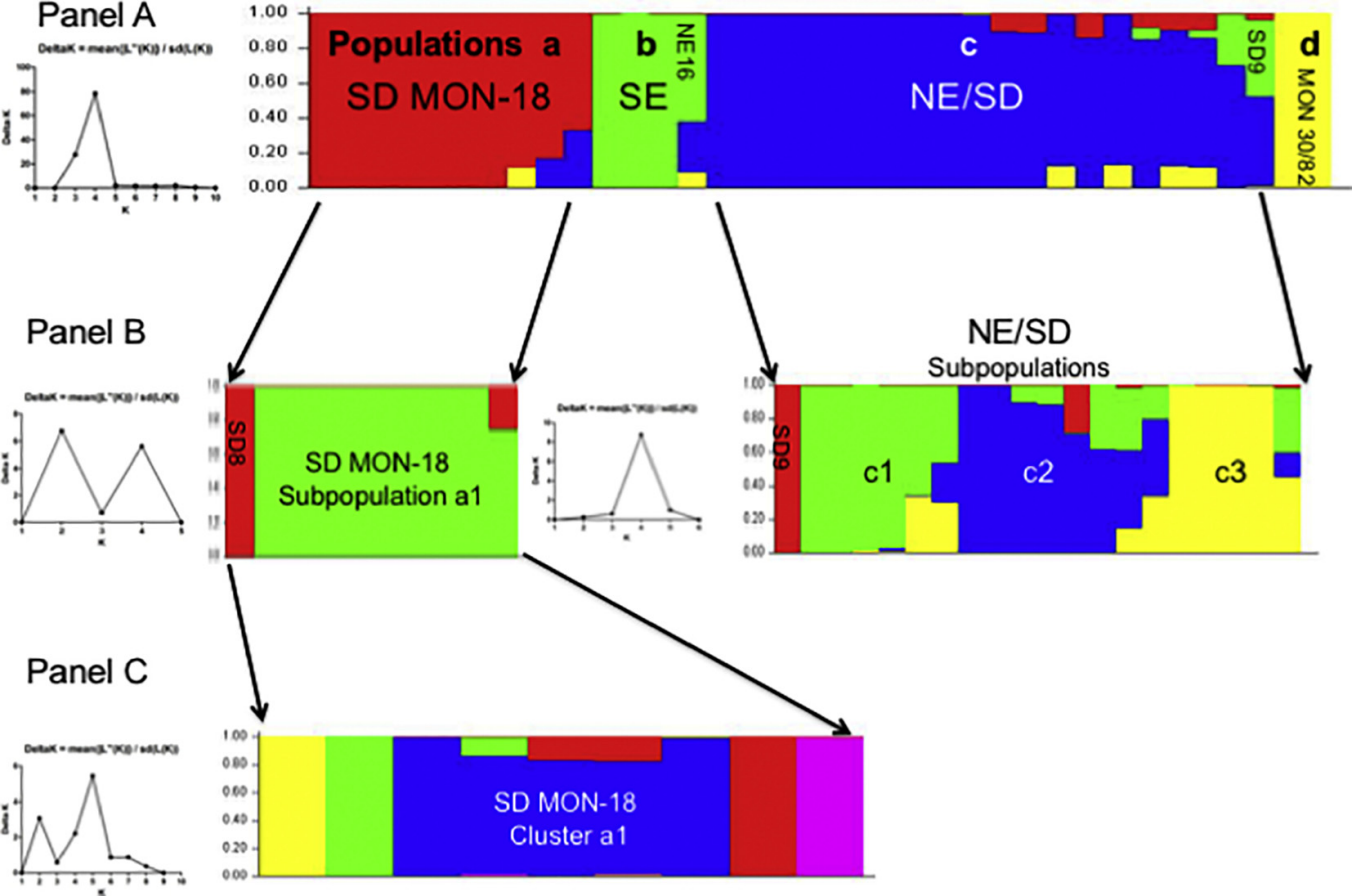
Population analysis of the 36 east African *Leishmania donovani* strains as determined by STRUCTURE (Pritchard et al., 2000) based on AFLP using six selective primers. A single vertical bar represents each strain with the different colors indicating to the fraction of genotype attributed by each population (Q). A single colour indicates that a strain belongs to only one genetic group. Population parameters were estimated by 10 Markov Chain Monte Carlo iterations after a burn-in of 100,000 simulations. Panel A. At K = 4 the strains group into four distinct populations which was confirmed by ∆K analysis, plot on left, using Structure Harvester (Earl and Vonholdt, 2012): a) SD MON-18 - all 10 Sudanese MON-18 strains; b) SE - the southern Ethiopian strains plus one atypical northern Ethiopian (NE) HIV/VL bone marrow strain (NE16); c) NE/SD - 17/18 NE strains and three of the SD strains: PKDL strain SD9 (MON-267), SD1 and SD2; and d) MON30/82 –PKDL strains SD3 and SD15 with zymodemes MON-82 and MON-30, respectively. Panel B. Further analysis of SD MON-18 and NE/SD by STRUCTURE identified additional subgroups. Subpopulation a1 contains all of the SD MON-18 strains except SD8, while the NE/SD population was divided into four subpopulations: c1 - NE1, NE4, NE6 - NE8 and SD2; c2 - NE2, NE3, NE5, NE9, NE10, NE17, NE18 and SD1; c3 - NE11 - NE15; and a fourth group containing the PKDL isolate SD9 (zymodeme MON-267). Panel C. Further analysis of SD MON-18 subpopulation a1. Cluster a1 contains SD6 SD7, SD10, SD11 and SD12.

The AFLP alleles for all the strains, except were also analyzed using Bayesian clustering by STRUCTURE (Pritchard et al., 2000). Only one technical control replicate for SD8 and NE2 was included in this analysis. Ten iterations were run for increasing values of K (1−10) using the default parameters. Four clusters were defined and stable at K = 4. Calculation of ΔK using Structure harvester (Earl and Vonholdt, 2012) confirmed this value (Fig. 3, panel A left). The population distribution observed using STRUCTURE is very similar to that seen in the Neighbor-net graph in Fig. 2 with the 36 *L. donovani* strains distributed among four major populations (Fig. 3, panel A right) as follows: a) the SD MON-18 group, which only contains SD strains with this zymodeme; b) the SE group, which contains the three SE strains examined, and an atypical NE HIV/VL bone marrow strain (NE16) that is genetically similar to SE strains based on k26–PCR (410 bp amplicon) (Zackay et al., 2013) and SNP analysis (Zackay et al., 2018); c) the NE/SD group that contains 17 out of 18 NE strains, and three SD strains. The latter includes the reference strains SD1 and SD2, and the PKDL strain SD9 with zymodeme MON-267; and d) a small group containing only the two PKDL strains belonging to either zymodeme MON-82 or MON-30. The two major populations, a and c, were analyzed separately by STRUCTURE to check for hidden structures within these groups. Two subpopulations (a1 and SD8) in population a containing the SD MON-18 strains, and four (c1 – c3 and SD9) in population c containing NE/SD strains were identified (Fig. 3, panel B). The c subpopulations contained the following strains: c1 - bone marrow (NE1, NE4 and NE6) and spleen strains from VL patients (NE7, NE8), as well as the reference strain SD2; c2 - spleen and bone marrow strains from VL patients (NE2, NE3, NE9 and NE5, NE10, respectively), strains from HIV/VL (NE17 and NE18) patients, and the reference strain SD1; c3 - spleen and skin strains isolated from the same two Ethiopian HIV/VL patients (NE11 - NE14), as well as the spleen HIV/VL strain NE15.

A third and last round of analysis was performed on all the SD/MON-18 strains in subpopulation a1. Nine iterations were run for increasing values of K (1–9) using the default parameters. Calculation of ΔK (Fig. 3, panel C left) using Structure harvester (Earl and Vonholdt, 2012) suggested the presence of additional substructures which included one major cluster a1 containing five strains. Each of the remaining strains (SD13, SD5, SD4 and SD14) grouped separately.

**Fig. 4:**
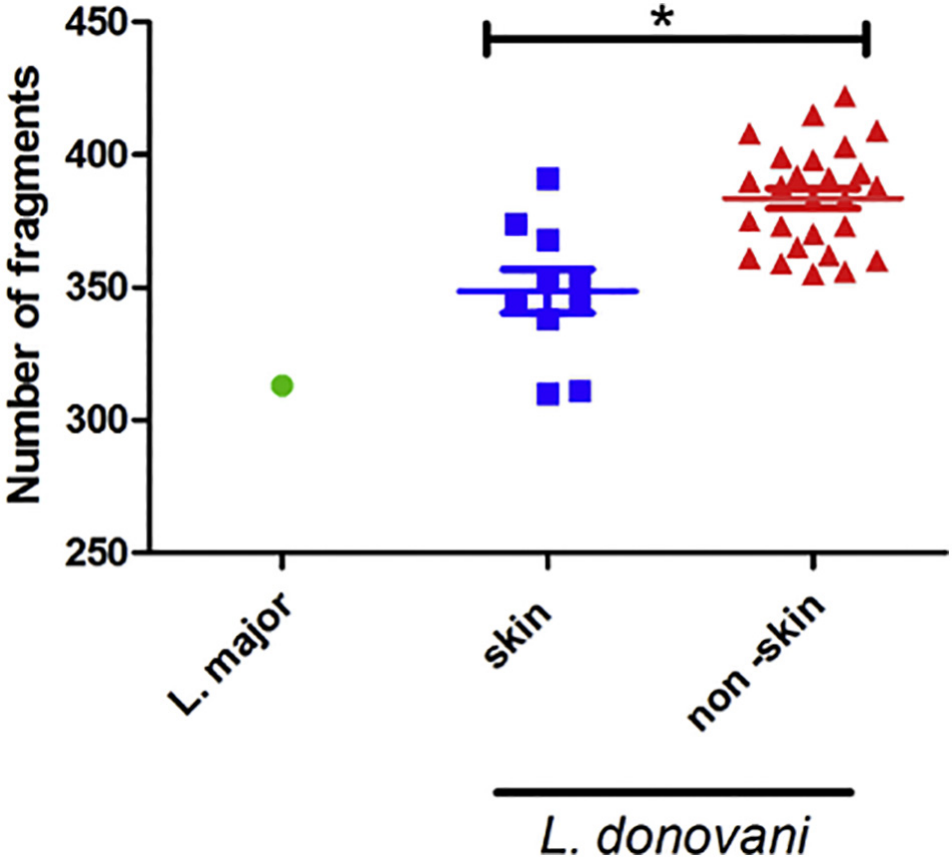
Number of AFLP alleles for skin and non-skin *Leishmania* strains. AFLP analysis using six selective primers was performed on 36 *L. donovani* and one *L. major* strains. The total number of polymorphic AFLP fragments was plotted according to tissue from which strain was cultured. Skin: 10 strains were isolated from the skin of northern Ethiopian HIV/VL and Sudanese PKDL patients. Non-skin: 26 strains were isolated from the spleen, bone marrow and lymph nodes of VL and HIV/VL patients from Sudan and Ethiopia. Skin isolates (average 10 strains = 348.6 ± 8.1), VL strains (average 26 strains = 383.5 ± 3.8). *unpaired *t*-test, *p* < 0.0001.

The total polymorphic AFLP allele number using 6 pairs of primers for each strain examined is plotted in Fig. 4. The skin group includes strains isolated from the skin of HIV/VL and PKDL patients, while the non-skin group includes strains isolated from the spleen, bone marrow or lymph nodes of VL patients from SD, SE & NE; and from HIV/VL patients in NE. Interestingly, skin isolates have significantly fewer polymorphic AFLP fragments (average 10 strains = 348.6 ± 8.1) than VL strains (average 26 strains = 383.5 ± 3.8) regardless of the year of the isolation or the place of origin (95% CI difference in means = 18.86 to 50.94, unpaired *t*-test, *p* < 0.0001). Further work is needed to confirm whether strains isolated from the skin show lower genetic complexity than strains isolated from internal organs.

### 3.2 Kinetoplast DNA sequences

**Fig. 5:**
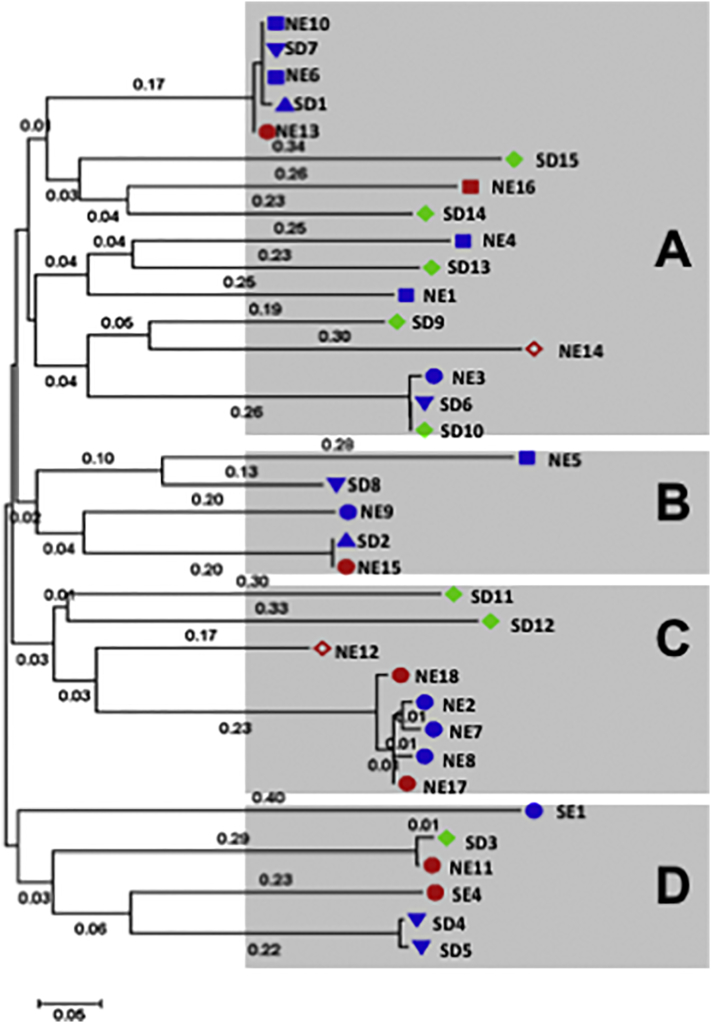
Phylogenetic tree of East African *Leishmania donovani* strains based on analysis of kDNA minicircle sequences. The sequences (GeneBank accession numbers KY950645 to KY950679) were aligned using Muscle algorithm with gaps, and the phylogenetic tree was built using the Maximum Likelihood method based on the Hasegawa-Kishino-Yano model (Hasegawa et al., 1985) using MEGA7 (Kumar et al., 2016) as described in Material and Methods. The tree is drawn to scale, with branch lengths measured in the number of substitutions per site (next to the branches). Strain origin: Northern (NE) or Southern Ethiopia (SE), Sudan (SD). Clinical presentation: VL – blue, HIV/VL co-infection – red, PKDL – green. Tissue source: skin isolates – full and empty diamonds, spleen isolates - circles, lymph node -inverted triangle, bone marrow isolates - squares, and no information - pyramid. Strains included in Group A – NE1, NE3, NE4, NE6, NE10, NE13, NE14, NE16, SD1, SD6, SD7, SD9, SD10, SD13 - SD15; Group B –NE5, NE9, NE15, SD2 and SD8; Group C – NE2, NE7, NE8, NE12, NE17, NE18, SD11 and SD12; Group D –NE11, SD3, SD4, SD5, SE1 and SE4. (For interpretation of the references to colour in this figure legend, the reader is referred to the web version of this article.)

kDNA minicircles are highly polymorphic (Rogers and Wirth, 1987), and both kDNA RFLP and sequencing have been used to examine *Leishmania* genotypes (Bhattarai et al., 2010; Cortes et al., 2006; Cuervo et al., 2004; Laurent et al., 2007). The complete minicircle was amplified using PCR primers to conserved regions, cloned and sequenced. The minicircle sequences were aligned and the distribution of the genotypes examined. The dendrogram based on analysis of minicircle kDNA sequences (Fig. 5) differed from that obtained by AFLP based on genomic DNA (Fig. 2). Unlike the latter case where the strains were distributed into major clades, SE and SD/NE, according to geographical origin or within the SD/NE population, by year of isolation, this was not observed using kDNA sequence analysis. Instead the strains were distributed in no particular order or pattern among four groups A - D. Group A was the most heterogeneous including sixteen strains isolated from all the tissues examined (spleen, lymph node, skin and bone marrow); from SD and NE; and from HIV co-infected (red), PKDL (green) and VL (blue) patients. Group B was comprised of five strains, three from NE and two from SD. The kDNA sequences of NE15 and the reference strain, SD2, are identical even though they were isolated 47 years apart. Group C contained eight strains the majority of which, six, were from NE. Interestingly, five of the strains in this group, isolated from spleens of VL patients (NE2, NE7, NE8, NE17 and NE18), have almost identical kDNA minicircle sequences (>98.3%). The remaining three strains were isolated from the skin (two PKDL and one HIV/VL co-infection), Finally, Group D contains 6 strains. This group is highly heterogenous containing strains from NE, SE and SD, as well as VL, PKDL and HIV/VL co-infected patients. No information describing the tissue source for the SD1 or SD2 strains was found in the literature, but they were likely isolated from spleen or bone marrow, and not skin.

**Fig. S2:**
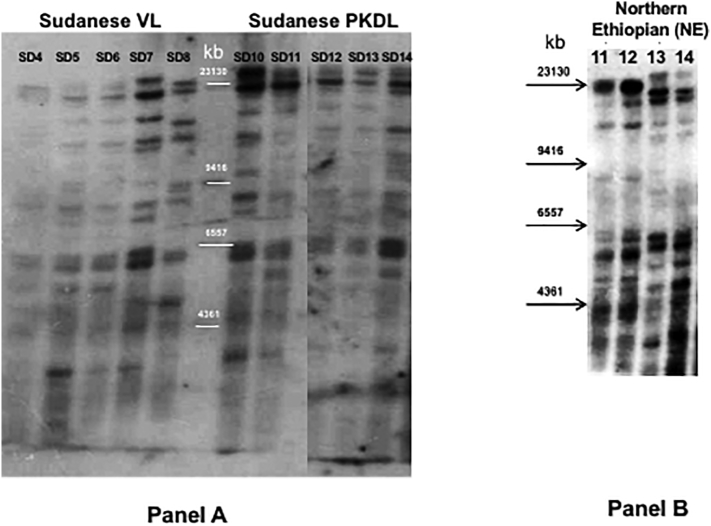
Southern blot of East African *Leishmania donovani* strains using a DNA probe (Ld28S) for the ζ and ε subunit regions of the 28S rRNA gene. Panel A: Sudanese VL or PKDL strains. Lanes 1–5: VL strains (SD4 – SD8); Lanes 7–11: PKDL strains (SD10 – SD14). Panel B; HIV/VL strains isolated from different organs, spleen (NE11 and NE13) or skin (NE12 and NE14), of patients GR363 (NE11 and NE12) and GR364 (NE13 and NE14).

Parasites isolated from skin or spleen of identical patients (NE11 and NE12; and NE13 and NE14) grouped in different branches in the kDNA minicircle tree, unlike the AFLP where all four strains clustered together (Fig. 2), and by Southern blotting where isolates from the same patient had identical profiles (Fig. S2, panel B).

### 3.3 Southern blot analysis

An earlier study suggested that Southern blotting using a DNA probe to the *L. donovani* 28S ribosomal RNA (28S rRNA) ζ and ε subunits could differentiate between parasites isolated from Indian VL (spleen) and PKDL (skin) patients (Sreenivas et al., 2004). A similar probe (Ld28S) was used to examine strains belonging to zymodeme MON-18 isolated from SD VL (lymph node) and PKDL (skin) patients (Fig. S2, panel A), as well as NE strains isolated from different tissues, skin and spleen, of the same patients (Fig. S2, panel B). Only minor differences in the Southern blotting patterns were observed among the SD parasites regardless of the clinical pathology, VL or PKDL. Indeed, with the exception of SD4 and SD8 (Panel A) the patterns looked almost identical, and differences observed for strain SD4 may be due to the low labeling intensity. In addition, no specific band or pattern, as previously suggested for Indian VL and PKDL strains (Sreenivas et al., 2004), was observed that differentiates between SD VL and PKDL strains. Interestingly, the VL strain SD8 also did not group with the SD MON-18 subpopulation a1 when analyzed by STRUCTURE (Fig. 3). Likewise, when matched strains, taken at the same time from different tissues in two HIV/VL NE patients (NE11 / NE12 and NE13 / NE14), were examined (Fig. S2, panel B), the patterns obtained for the spleen and skin isolates from each patient appeared essentially identical, while the banding patterns between parasites from the two patients were different.

## 4 Discussion

In this study, 36 East African *L. donovani* strains were examined by AFLP generating 411 polymorphic markers that were used for analysis of genetic diversity. The population distribution obtained by Neighbor-net (Fig. 2) and STRUCTURE (Fig. 3) analysis of the AFLP alleles was similar. The SE strains, using both methods, consistently grouped together and separate from the SD/NE strains, which were split into several subpopulations. Geographic separation of the SE and SD/NE *L. donovani* strains is congruent with other molecular genetic techniques such as MLMT (Gelanew et al., 2010b; Kuhls et al., 2007) and SNP analysis of whole genome sequences (Zackay et al., 2018). This also supports previous AFLP results using a limited number of East African strains (Odiwuor et al., 2011). This finding is perhaps not surprising, as the biotopes and vectors that transmit these parasites are different between the two regions. In addition, patients with VL in NE/SD and SE were shown to respond differently to drug treatment.

STRUCTURE analysis divides the East African panel of *L. donovani* from NE and SD into three distinct populations a, c and d as seen in Fig. 3. These populations also appear as distinct groups in the Neighbor-net graph (groups 1–3) and are supported by high bootstrapping values. The SE strains also group as a separate population, b, by STRUCTURE analysis. Subpopulation a1 contains all the Sudanese MON-18 isolates. These strains were isolated from both VL and PKDL patients in the village of Barbar El Fugara during a two-year period. The VL and PKDL isolates were interspersed suggesting that parasites causing these different clinical symptoms are highly similar. Population c contains all of the NE strains, except for NE16 that appeared to group with the SE strains. The latter isolate was more similar to SE strains when genome-wide SNP analysis of Ethiopian *L. donovani* strains was carried out (Zackay et al., 2018). The further distribution of population c among three subpopulations (c1 - c3) was supported by the almost identical grouping of these strains by the Neighbor-net analysis into three clusters 3a, 3b and 3c. Interestingly, the two reference strains SD1 and SD2 also group with the NE isolates in population c, similar to what is observed in the Neighbor-net graph. Finally, population d consists of only two SD PKDL strains, SD3 (MON-82) and SD15 (MON-30) and is supported by a 100% bootstrap value (Group 1, Fig. 2).

Analysis of the AFLP markers failed to group the East African *L. donovani* strains according to clinical symptoms, since PKDL and other skin isolates (NE12 and NE14) were interspersed among the VL isolates from lymph nodes, bone marrows and spleens. Lack of grouping by clinical symptoms was also found when *L. donovani* strains from East Africa were examined by MLMT and MLST (Baleela et al., 2014). However, it is interesting to note that the number of polymorphic AFLP fragments in skin strains is lower than for the non-skin strains, regardless of the year or site of isolate origin. AFLP allele number was also lower in *L. (V.) peruviana* which only causes CL compared to the *L. (V.) braziliensis* which causes both CL and the mucocutaneous form (Odiwuor et al., 2012). This simpler genetic profile may arise from (i) changes in enzyme restriction site distribution due to point mutations; and (ii) gene rearrangements caused by amplification/deletion of repetitive sequences (Lighthall and Giannini, 1992) or DNA recombination (Liu et al., 1992). Thus, the finding that *L. donovani* skin isolates have lower number of polymorphic AFLP fragments than non-skin isolates warrants further investigation.

kDNA minicircle sequencing was also used to examine genetic diversity of all the East African *L. donovani* strains. A high degree of polymorphism was observed using this approach where the isolates were distributed among four different groups (A – D). Unlike with AFLP, no connection to geographic origin was observed and the SD, NE and SE strains were mixed among most of the clusters. Moreover, there appeared to be no association between parasite tissue origin and group. The PKDL and other skin isolates were distributed throughout three of the four clusters. While only one kDNA minicircle sequence per strain was examined in this study, each parasite strain actually contains multiple, varying minicircle sequence classes (Simpson et al., 2015). In addition, the percentage of individual minicircle classes is known to change during continuous long-term culture potentially affecting analysis of leishmanial genetic diversity (Simpson et al., 2000). Further studies, using Next Generation Sequencing of PCR products directly amplified from lesion biopsy material, would allow for accurate, in-depth evaluation of different kDNA minicircle classes and their sequences without need to culture parasites.

**Fig. S3:**
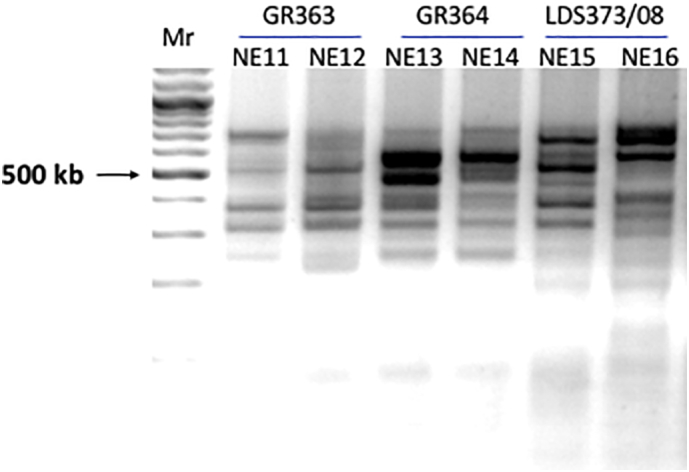
Analysis by kDNA PCR-RFLP of HIV/VL strains isolated from different organs of the same patient. kDNA minicircles from three patients were amplified by PCR using the primer pair Uni21 and Lmj4 (Anders et al., 2002) and purified as described in Material and Methods. The purified products were digested overnight with HaeIII and separated by electrophoresis on 3% NuSieve 3:1 Agarose (Cambrex Bio Science Rockland, Inc.) gels. Lanes: Mr. - 100 kb marker; GR363 NE11 – spleen and NE12 – skin; GR364 NE13 – spleen and NE14 – skin; LDS373/08 NE15 – spleen and NE16 – bone marrow.

Interestingly, kDNA minicircle sequencing (Fig. 5) discriminated between pairs of isolates taken from the skin and spleen of the same HIV/VL co-infected patient, unlike AFLP and Southern blotting where each pair was essentially identical. While we can't exclude the possibility that the differences observed in kDNA minicircle sequences for each skin/spleen pair originates from subcloning the PCR products prior to sequencing, kDNA PCR-RFLP analysis shows different patterns for the spleen (NE11 and NE13) and skin (NE12 and NE14) strains of each patient indicating that these strains contain kDNA minicircle classes with different sequences (Fig. S3). A similar result was seen when spleen (NE15) and bone marrow (NE16) isolates from the HIV/VL co-infected patient LDS373/08 were analyzed by kDNA PCR-RFLP. Interestingly, cluster analysis, based on chromosome copy number, grouped the skin and spleen strain pairs (NE12 and NE14; NE11 and NE13, respectively) separately based on tissue source (Zackay et al., 2018). While the presence of mixed tissue infections and subsequent selection of distinct lines by culture passage of primary isolates prior to analysis cannot be ruled out, these results suggest that parasite residence in the distinct tissue environments can lead to selection of different kDNA minicircle classes. Similarly, kDNA PCR-RFLP was also shown to differentiate between pairs of cutaneous and mucosal *L. (Viannia)* strains isolated from the same patients (Cuervo et al., 2004). Searching the literature, we have found only one report indicating the relationship between kDNA and tropism where the authors cloned a kinetoplast DNA mini-fragment from *Leishmania* strains that hybridized only to strains from PKDL patients and not to strains from VL patients (Das Gupta et al., 1991).

On the other hand, the strain pair NE15 and NE16 isolated at the same time from spleen and bone marrow, respectively, of an HIV/VL co-infected patient, appears to be the result of a mixed infection showing different genotypes by AFLP, kDNA sequencing, whole genome SNP analysis and k26 –PCR (this study; Zackay et al., 2013; Zackay et al., 2018). Mixed infections in a Brazilian HIV/VL co-infected patient have been described where parasites isolated from the bone marrow and skin were typed as *L. infantum* MON-1 and *L. donovani* MON-2, respectively (Scaglia et al., 1996). Similarly, *L. infantum* parasites with two different zymodemes, MON-1 and MON-77, were isolated simultaneously from the skin and lymph nodes of a dog with diffuse leishmaniasis (Pratlong et al., 1989).

Finally, our results agree with the previous studies investigating strains collected from the same outbreak that occurred in the 1990s in the village Barbar El Fugara (Dereure et al., 2003; El-Safi et al., 2002; Pratlong et al., 2001; Rougeron et al., 2011). Of the seven zymodemes circulating in the VL endemic area in eastern SD, four zymodemes were isolated from PKDL and VL patients (MON-18, MON-30, MON-267 and MON-82) suggesting no association between a particular zymodeme and the risk of developing PKDL (Dereure et al., 2003; Pratlong et al., 2001; Rougeron et al., 2011; Zijlstra and El-Hassan, 2001). Similarly, when PCR single-strand conformation polymorphism was used to examine the SD strains, no correlation was found with the clinical manifestations of VL and PKDL (El Tai et al., 2001). Analysis of strains from Barbar El Fugara by MLMT or MLST showed that parasites from both VL and PKDL patients clustered in the same branches of the tree (Baleela et al., 2014; Rougeron et al., 2011). Similarly, AFLP analysis grouped most of the VL and PKDL strains from this focus together.

Even though we attempted to limit possible confounding factors (zymodeme, location, years collected) for the Sudanese strains examined, information on the development of PKDL in the VL patients, and the corresponding parasite strains, are lacking. Thus, it is very possible that unidentified genetic factors play a role in PKDL development. Comparison of paired VL and PKDL strains sequentially collected from individual patients, as well as strains from VL patients which never develop PKDL, by techniques such as whole genome sequencing is needed. Unfortunately, such paired strains are frequently difficult to acquire, and most results reported to date suggest that the development of PKDL is correlated with other factors (Dereure et al., 2003; El-Safi et al., 2002).

To our knowledge, this is the first time that AFLP and kDNA minicircle sequencing was used to differentiate between East African *L. donovani* strains isolated from different organs. AFLP has proved to be a powerful technique for inferring phylogenetic relationships within a *Leishmania* species isolated from different geographic regions, but similar to MLMT and MLST, was not able to identify genetic associations with the clinical outcome. On the contrary, kDNA minicircle sequences yielded a completely different phylogenetic distribution of the strains, which was not associated with geographic origin. No correlation between the two techniques was observed. Further analysis of additional East African *L. donovani* strains from different tissue sources and from patients with varying clinical presentations are needed to substantiate these findings, as only a limited number of strains (36) from restricted geographic regions, and years of isolation were examined in this study. However, these preliminary results pave the way for more research aimed at understanding the genetic determinants and the underlying biology of tropism in this parasite.

The following are the supplementary data related to this article.

Supplementary material 1 Image 1

Supplementary material 2 Image 2

Supplementary material 3 Supplementary material
