# Supplementary material for: Analysis of genetic polymorphisms and tropism in East African Leishmania donovani by Amplified Fragment Length Polymorphism and kDNA minicircle sequencing
Source: Infect Genet Evol. 2018 Nov;65:80–90. doi: 10.1016/j.meegid.2018.07.016 (PMC6218636; doi:10.1016/j.meegid.2018.07.016)
Supplement: Supplementary file 2 — Supplementary material 2 [file mmc2.pdf]

Table S2: List of primers and double-stranded adaptors used for AFLP

Type of sequences	Name	Sequence
**Double-strand adaptors**	*Pst*I-adaptor	5'-CTCGTAGACTGCGTACATGCA-3'<br>||| || || | ||| || | 3'- CATCT GACGCATGT-5'
	*Taq*I-adaptor	5'-GACGATGA GT CC TGAG-3'<br>|||| | | || || || 3'-TACT C AGG ACTCGC-5'
**Pre-selective primers**	PstI+0	GACTGCGTACATGCAG
	TaqI+0	GATGAGTCCTGAGCGA
***Taq*I-specific selective primers**	Taq+AA	GATGAGTCCTGAGCGAAA
	Taq+AC	GATGAGTCCTGAGCGAAC
	Taq+AG	GATGAGTCCTGAGCGAAG
***Pst*I-specific selective primers**	PstI+AG	**6FAM**-GACTGCGTACATGCAGAG
	PstI+CC	**NED**-GACTGCGTACATGCAGCC
	PstI+GT	**PET**-GACTGCGTACATGCAGGT
	PstI+TG	**VIC**-GACTGCGTACATGCAGTG

The six primer pairs used to draw the phylogenic tree in fig 1: Taq+AA & PstI+AG, Taq+AC & PstI+AG, Taq+AA & PstI+TG, Taq+AC& PstI+TG, Taq+AG & PstI+TG and Taq+AC & PstI+CC
