# Supplementary material for: Analysis of genetic polymorphisms and tropism in East African Leishmania donovani by Amplified Fragment Length Polymorphism and kDNA minicircle sequencing
Source: Infect Genet Evol. 2018 Nov;65:80–90. doi: 10.1016/j.meegid.2018.07.016 (PMC6218636; doi:10.1016/j.meegid.2018.07.016)
Supplement: Supplementary file 3 — Supplementary material 3 [file mmc3.docx]

**Supporting Data**

**Material and Methods**

**Probe** **preparation for use in Southern blotting:**

A DNA probe (Ld28S) to the **ζ** and **ε** subunit regions of the *L. donovani* (MHOM/SD/00/Khartoum) 28S rRNA gene (accession no. [AF115465](http://www.ncbi.nlm.nih.gov/nuccore/5019758)) was prepared by PCR using the following primers, f28S (5’- CAC AAC CTT TTC CGT GG TTT - 3’) and r28S (5’- CCC CCT CCT ATA TTT GTG TCC - 3’), respectively. These primers amplify a fragment spanning the region from 627 to 1472 bp of the 28S rRNA gene. Genomic DNA (50 ng) was added to a reaction mixture (25-μl final volume) containing High Yield ready mix (Syntezza Bioscience^®^) and both primers (4 μM). The DNA was initially denatured at 95 °C for 5 min, followed by 31 cycles of denaturation at 95 °C for 30 s, annealing at 58 °C for 30 s, and elongation at 72 °C for 75 s. A final elongation step at 72 °C for 10 min was carried out after the last cycle. The PCR product was cleaned using Wizard SV Gel and PCR Clean-Up system (Promega^®^) and the 846 bp amplicon cloned into pGEM-T Easy Vector (Promega^®^) according to the manufacturer's instructions. DH5α cells were transformed using heat shock for 45 s at 42 °C, and grown in LB medium. Plasmid DNA was purified using the HiYield™ Plasmid Mini Kit (RBC Bioscience Corp. ^®^), and the insert sequenced.

The Ld28S probe was labeled with DIG-dUTP (Roche Applied Science^®^) by PCR using the f28S and r28S primers, and pGEM-T Easy Vector DNA containing the insert as template. Plasmid DNA (1 to 2 ng) was added to a reaction mixture (50-μl final volume) containing primers (2 μM), JumpStart™ Taq DNA Polymerase with MgCl_2_ (2.5 U, Sigma Aldrich^®^), PCR DIG labeling mix (0.2 mM), MgCl_2_ (3 mM) and 5 μl of JumpStart™ Taq DNA Polymerase 1× buffer (Sigma Aldrich^®^). The PCR reaction was performed as described above. Purity of the DIG-labeled probe was examined by agarose gel electrophoresis (1.5%), and the probe was stored at −20 °C until use.

**Southern blotting:**

Purified DNAs (5 μg) were digested for 2 hours at 60 °C with MvaI (4 U/μg of DNA; new England BioLabs Inc. ^®^). Complete digestion was ensured by incubation with an equivalent amount of enzyme overnight. Digestion products were separated by 0.7% agarose gel electrophoresis and Southern blotting using alkaline transfer was carried out using the DIG-labeled probe (Detector HRP Chemiluminescent Blotting Kit, KPL^®^). Following UV crosslinking (UV Stratalinker 1800; Stratagene^®^) the membrane was incubated in DIG Easy Hyb (Roche Applied Science^®^) containing ssDNA (200 µg /mL) for 60 min at 42 °C and the DIG-labeled probe hybridized to the membrane overnight at 42 °C. Following hybridization, the blot was treated as recommended by the manufacturer and then incubated with the sheep anti-DIG alkaline phosphatase antibody (1/10,000 dilution in blocking solution; Roche Applied Science^®^). Probe hybridizing to the DNA on the blot was detected following incubation of the blot with the substrate CDP-Star (Roche Applied Science^®^) for 5 min, and exposure to X-ray film.
